# Magnetic resonance imaging in diagnosis of indeterminate breast (BIRADS 3 & 4A) in a general population

**DOI:** 10.1186/s13244-021-01098-z

**Published:** 2021-10-21

**Authors:** Liliana Hernández, Gloria M. Díaz, Catalina Posada, Alejandro Llano-Sierra

**Affiliations:** 1 Grupo de Investigación del Instituto de Alta Tecnología Médica (IATM), Ayudas Diagnósticas Sura, Medellín, Colombia; 2 MIRP Lab–Parque i, Instituto Tecnológico Metropolitano, Medellín, Colombia; 3 Universidad CES, Medellín, Colombia

**Keywords:** Breast cancer, Indeterminate lesions, Magnetic resonance imaging, Mammography, Ultrasonography

## Abstract

**Objective:**

Currently, mammography and ultrasonography are the most used imaging techniques for breast cancer screening. However, these examinations report many indeterminate studies with a low probability of being malignant, i.e., BIRADS 3 and 4A. This prospective study aims to evaluate the value of breast magnetic resonance imaging (MRI) to clarify the BIRADS categorization of indeterminate mammography or ultrasonography studies.

**Methods:**

MRI studies acquired prospectively from 105 patients previously classified as BIRADS 3 or 4A were analyzed independently by four radiologists with different experience levels. Interobserver agreement was determined by the first-order agreement coefficient (AC1), and divergent results were re-analyzed for consensus. The possible correlation between the MRI and the mammography/ultrasound findings was evaluated, and each study was independently classified in one of the five BIRADS categories (BIRADS 1 to 5). In lesions categorized as BIRADS 4 or 5 at MRI, histopathological diagnosis was established by image-guided biopsy; while short-term follow-up was performed in lesions rated as BIRADS 3.

**Results:**

Breast MRI was useful in diagnosing three invasive ductal carcinomas, upgraded from BIRADS 4A to BIRADS 5. It also allowed excluding malignancy in 86 patients (81.9%), avoiding 22 unnecessary biopsies and 64 short-term follow-ups. The MRI showed good diagnostic performance with the area under roc curve, sensitivity, specificity, PPV, and NPV of 0.995, 100%, 83.5%, 10.5%, and 100%, respectively.

**Conclusions:**

MRI showed to be useful as a problem-solving tool to clarify indeterminate findings in breast cancer screening and avoiding unnecessary short-follow-ups and percutaneous biopsies.

## Key points

- MRI exclude malignancy in 81.9% of BIRADS 3 and 4A lesions.
- MRI identified three Invasive Ductal Carcinomas by upgrade lesions from BIRADS 4A to 5.
- BIRADS scoring of MRI examinations shows moderate/substantial interobserver agreement between radiologists.
- MRI has good diagnostic validity in patients with previous examinations categorized as BI-RADS 3 or 4A.
- MRI can be considered a tool to clarify indeterminate findings in breast cancer screening.

## Introduction

Breast cancer is the most frequently diagnosed cancer in women and the leading cause of death by cancer among women worldwide [1]. X-ray mammography remains the standard screening method for detecting it in women over 40 years, which has been demonstrated to reduce breast cancer mortality in percentages ranging from 30 to 70% [2]. However, it has variable sensitivity, specificity, and predictive values, which affect its confidence [3]. Likewise, ultrasonography is also used as a screening tool, specially indicated for young and lactating or pregnant women, and as a supplement to mammography screening in women with heterogeneously or extremely dense breasts. This technique presents limitations to detect small lesions and differentiate the cysts with dense contents from solid lesions and be a user-dependent technique [4]. Therefore, imaging alternatives, such as tomosynthesis, contrast ultrasonography, elastography, and magnetic resonance imaging (MRI), have been proposed to replace mammography and ultrasonography as a population screening method [5–8]. Breast MRI is considered the most sensitive method for detecting breast cancer without the use of ionizing radiation; hence, it has been proposed as an effective screening alternative in the high-risk population [9–12].

Additionally, the American College of Radiology (ACR) developed the Breast Imaging-Reporting and Database System (BIRADS), which aims to standardize the breast lesion descriptions to reduce the Interobserver variability, to ease the communication with the clinician, and improving the management of the patient. These descriptions aid physicians in deciding the BIRADS category (0 to 6), a value that determines the probability of malignancy and the final management recommendation [13]. BIRADS 3 and 4A categories define most probably benign lesions with malignancy risk less than 2 and 10%, respectively, for which it is advised short-term follow-up imaging or biopsies, which can increase costs for the health system morbidity, and patient anxiety [14, 15].

In cases of indeterminate mammographic and ultrasonographic findings, i.e., lesions rated as BIRADS 3 or 4A, the use of other imaging modalities as “problem-solving” tools has been proposed. Regarding MRI, the American College of Radiology practice guidelines include them under the category of lesion characterization; breast MRI may be indicated when other diagnostic imaging examinations or physical examinations are inconclusive for the presence of breast cancer, and biopsy cannot be performed [16]. However, some studies have suggested that MRI imaging may help the clinician manage BIRADS 3 and 4A lesions and eliminate unnecessary biopsies [16–22].

The purpose of this single-center study was to prospectively investigate the usefulness of an MRI examination for evaluating lesions detected in mammography or ultrasonography screening that were categorized as BIRADS 3 or 4A from a group of women from a general Latin American female population.

## Materials and methods

### Study design and population

A prospective study approved by the institutional research and ethical committee was performed between June 2019 and March 2020. The technical staff of Ayudas Diagnósticas Sura (Medellín, Colombia) identified consecutively the patients who were categorized as BIRADS 3 or 4A on mammography and ultrasonography examinations. The identified patients were invited to participate in this prospective study. Those that accepted and met the inclusion criteria were scheduled to undergo the MRI examination following the institutional protocol. Informed consent was obtained from each patient.

Patients were considered eligible when they were over 18 years old and had examinations categorized as BIRADS 3 or 4A (ACR BIRADS® Atlas Fifth Edition) at mammography, ultrasonography or both during the last year. Patients with pacemakers, nonremovable drilling at the nipple or other devices in the chest wall, unable to keep upright immobility, claustrophobic or allergic to the contrast medium; or with a confirmed diagnosis of breast cancer, or history of carcinoma in situ, were excluded. All patients underwent the same MRI protocol, regardless of the finding for which they were referred.

### Imaging technique

The mammography and ultrasonography examinations were acquired and interpreted in service outside the institutions participating in this research. The radiological reports were requested from the participants during recruitment and archived for later use. For this reason, there was no control or follow-up on the acquisition or interpretation protocols of those examinations. All patients underwent a breast MRI protocol with a 1.5 T Philips resonator with a 7-channel breast dedicated coil. The patient was in the prone position. Examinations were scheduled on the second week of the menstrual cycle in premenopausal women, and no scheduling limitations were defined for postmenopausal women. The MRI protocol is described in Table 1. It encompassed one Axial T1-weighted non-fat-saturated sequence, followed by an Axial diffusion-weighted sequence with B0 and B 800 factors. For the dynamic contrast enhancement assessment, one unenhanced fat-suppression T1 sequence and six volumetric SPIR T1W High-Resolution Isotropic Volume Examination (THRIVE) sequences were acquired after the injection of meglumine gadoterate at a dose of 0.2 mL/kg (0.1 mmol/kg).

**Table 1:** Technical specifications of the breast magnetic resonance imaging protocol

	Axial T1W VISTA	DWI** B0 and B800	T1 THRIVE***1	Axial T2 VISTA	Coronal STIR****
Repetition time	7,5	7112	6.8	7010	10,000
Echo time	4,6	70	3.3	80	80
FOV* (mm)	280 × 368x180	300 × 400x198	300 × 337x156	280 × 370x180	299 × 372x200
Acquisition matrix	352 × 459	120 × 157	252 × 280	351 × 463	232 × 241
Acquisition time	3:44	2:36	6:07	3:16	4:40
Pre-contrast acquisitions	1	1	1	1	1
Post-contrast acquisitions			5 (60.1 s/seq.)		
Post-processing		ADC map	Subtraction		

*Field-of-view

**Diffusion-Weighted Images

***T1W High-Resolution Isotropic Volume Examination

****Short tau inversion recovery

The contrast medium was injected with an injector at a 2.5 ml/s rate with yelco infusion number 20. Subsequently, an Axial T2-weighted fast spin-echo (3D VISTA) without fat saturation and a coronal STIR sequence were acquired. When the patient had breast implants, the short tau inversion recovery (STIR) sequence was replaced by a T2 coronal fat suppression 3d VISTA to better contrast with the silicone (FOV 300 × 364 × 200, matrix 252 × 256, repetition time 7010, echo time 80, duration 3:16 min). Finally, the imaging technologist performs the subtraction of the dynamic sequences, obtains the color perfusion maps, and generates the Apparent Diffusion Coefficient (ADC) map. The images are sent to the PACS and the Invivo post-processing station for future interpretation.

### Image interpretation

MRI examinations were initially prospectively interpreted by one breast imaging radiologist (R1) with 12 years of experience interpreting breast MRI examinations. A computer-aided diagnosis (CAD) program (DynaCAD Philips, Inc.) was available for breast interpretation; however, its use was decided at the discretion of the interpreting radiologist. Then, all examinations were retrospectively and independently analyzed by three radiologists with 10 (R2), 5 (R3) and less than one (R4) years of experience (caseload ranging between 100 and 200 breast MRI examinations per year). The less experienced radiologist (R4) was one experienced mammogram reader (> 10 years) who was trained to interpreting the MRI scans. These radiologists were blinded to any concept of the others.

According to the location, size, and morphological characteristics, each radiologist evaluated a possible correlation between the MRI and mammography/ultrasonography findings reported by previous examinations. Additionally, radiologists evaluated the mammary tissue composition, the background physiological enhancement, the uptake asymmetry, the positive findings, and morphological and kinetic characteristics; they also determined the presence of tissue restriction given by the ADC value, the associated and incidental findings. According to the fifth version of the BIRADS lexicon, breasts were classified as A, B, C or D density categories; and minimal, mild, moderate, or marked background parenchymal enhancement; for the positive findings, in the case of the masses, the shape, margins and internal characteristics were described; and in the case of non-mass enhancements, the distribution and internal features were described. Post-gadolinium kinetic curve analysis was referred to as persistent, plateau, or wash-out pattern. Additionally, the associated findings evaluated the involvement of the skin or muscles and lymphadenopathy in internal or axillary mammary chains.

Once MRI findings have been identified, radiologists assign a category according to BIRADS fifth edition, based on the imaging features and other available information as previous breast imaging studies and the patient’s clinical history. Following institutional practice, radiologists were asked to follow the Kaiser scoring system. It is a simple decision rule-based flowchart that guides readers to a clinical decision about the risk of malignancy (scores from 1 to 11) by characterizing five specific diagnostic criteria, i.e., root sign(present/ absent), delayed enhancement curve type (persistent/ plateau/ wash-out), margins (smooth/ irregular), internal enhancement pattern (inhomogeneous/ homogeneous) and edema (diffuse ipsilateral or perifocal/ absent or diffuse bilateral) [23, 24]. The resulting score is translated into the BI-RADS categories that are finally reported [25]. Nevertheless, final categorizations were done at the discretion of the interpreting radiologist. The results were stored in a referential database to facilitate future consultation.

Divergent results were re-analyzed by the most experienced reviewers in consensus. According to the BIRADS lexicon, breasts with normal or benign findings fall into BIRADS 1 or 2 categories and continue the usual screening process; BIRADS 3 lesions are those that are probably benign, with a risk of malignancy less than 2%. These lesions require a short-term follow-up for up to two years to confirm their progress, stability, or regression. BIRADS 4 lesions have attributed risk of malignancy between 2 and 95%, and like BIRADS 5 lesions, which carry a risk of malignancy greater than 95%, they are always biopsied.

### Reference standards

All patients with MRI findings categorized as BIRADS 4 or 5 underwent biopsy. Histologic sampling was performed under ultrasonography or mammography imaging guidance depending on which method was best suited to locate and access the lesion. Patients with MRI examinations classified as BIRADS 3 underwent short follow-up, by at least one year, with mammography, ultrasonography, or MRI to check the stability of the findings. Patients with MRI examinations classified as BIRADS 2 were requested to perform clinical examinations. In cases that were required, new imaging tests were also performed.

### Statistical analysis

Considering BI-RADS 3 to 5 as positive findings and 1 and 2 as negative, the sensitivity, specificity, positive predictive values (PPV) and negative predictive values (NPV) with 95% confidence intervals (CI) were calculated. Overall accuracy was evaluated based on the receiver operating characteristic (ROC) analysis and the area under the curve (AUC) calculation.

On the other hand, the Interobserver agreement was calculated using the generalized kappa [26] and the first-order agreement coefficient (AC1) proposed by Gwet [27]. As Wongpakaran et al. [28] shown, it is not affected by the prevalence of the phenomenon under study. Breast cancer tumors were considered malignant, and all other histologic diagnoses and stable follow-up were considered as the absence of malignancy.

## Results

### Study population

**Fig. 1:**
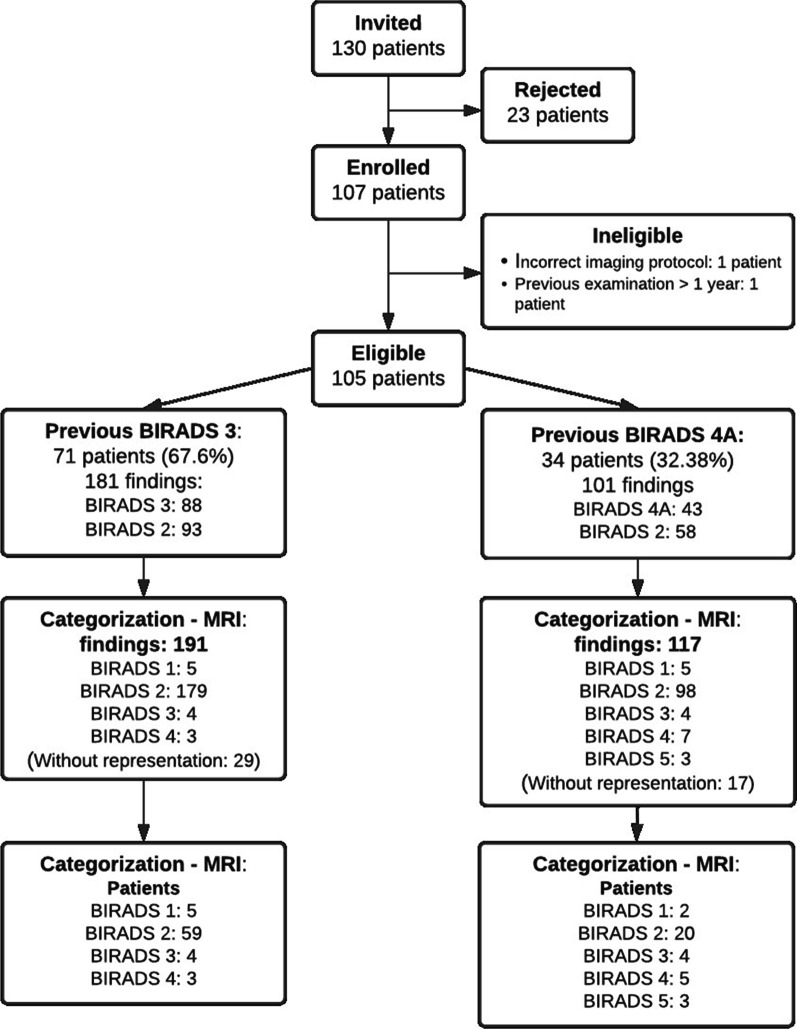
Patient selection flowchart and final findings stratified by BIRADS categories

Figure 1 provides the study patient selection flowchart. Between June 2019 and January 2020, 130 women with a previous breast image examination (mammography or ultrasonography) ranked as BIRADS 3 or 4A were invited to participate in this study. A total of 107 subjects agreed to participate, but two were determined to be ineligible; the first was due to previous examination results being larger than one year, and the second was due to errors in the acquisition protocol. Thus, 105 eligible patients were finally enrolled; 71 (67.62%) of them presented examinations categorized as BIRADS 3 and 34 (32.38%) as BIRADS 4A. Previous examinations were ultrasonography for 81 patients (82.85%), mammography for 11 patients (10.47%), and both ultrasonography and mammography for 13 patients (12.38%). A total of 282 findings were reported in previous examinations, 181 (64.18%) in examinations classified as BIRADS 3, and 101 (35.81%) in BIRADS 4A exams. All subjects underwent a Breast MRI examination before continuing with defined management, i.e., imaging follow-up or percutaneous biopsy. Time elapsed between the previous imaging and the MRI was 155.6 ± 117.5 (mean ± Standard Deviation) and 41.8 ± 26.79 days for previous BIRADS 3 and BIRADS 4A exams, respectively.

All patients were Colombian women, ranging from 20 to 70 years old with a mean age of 42.71 ± 11.15. Table 2 presents a summary of the demographic data of participants. Among 25 (23.8%) patients with previous histological proven benign findings, 18 were fibroadenoma, one an adenosis, one a cystic fibrosis, one a fibrocystic mastopathy, one a ductal hyperplasia, one a fat necrosis, and two were epidermal inclusion cysts. Two patients also had a history of breast cancer greater than five years. (in situ ductal carcinoma). Regarding family history, nine patients had a first-degree relative (mother), 11 had a second-degree relative (six grandmothers and five sisters), and two had a history of both first and second-degree relative (mother and grandmother). There were 68 premenopausal (64.8%) and 38 postmenopausal (35.2%) patients. For postmenopausal patients, the mean years past since the last menstruation was 8.4 ± 6.01 (range, 1–26 years), three patients were subjected to hysterectomy.

**Table 2:** Baseline demographics in the cohort of 105 subjects

Parameter	Result
*Age*	
Mean ± SD*	42.71 ± 11.1
Range	(20–70)
*Menarche*	
Mean ± SD	12.9 ± 1.8
Range	(11–18)
*Previous histological results*	
Benign	25 (23.8%)
No	80 (76.2%)
*Family history of breast cancer*	
1st grade	9 (8.6%)
2nd grade	11 (10.5%)
1st + 2nd grade	2 (1.9%)
No	83 (79%)
*Personal history of breast cancer*	
Yes	2 (1.9%)
No	103 (98.1%)
*Parity*	
Nulliparous	32 (30.5%)
1 birth	32 (30.5%)
2 births	32 (28.5%)
3 births	32 (10.5%)
*Menopause*	
Premenopausal	68 (64.8%)
Postmenopausal	37 (35.2%)

*Standard deviation

### MRI findings

After the consensus of radiologists, a total of 308 findings were identified at the MRI examinations. From these, 191 were found in examinations performed on patients enrolled as BIRADS 3, and the other 117 were found at the MRI exams of patients with previous images categorized as BIRADS 4A. Table 3 shows a summary of the main imaging features of those findings. From 105 examinations, 11.4% showed minimal background parenchymal enhancement (BPE); 36.2%, mild; 34.3%, moderate; and 18.1%, marked enhancement. In the group with minimal BPE, only one examination was classified as BIRADS 3 due to a suspicious nodule not being seen in previous examinations. The other BIRADS 3, 4 or 5 examinations corresponded to mild (44%), moderate (33%) and marked (17) BPE. On the other hand, breast density was high (ACR C or D) in a total of 81 patients (77.1%), which was expected because they had BIRADS 3 and 4A examinations. Less than half of the described findings showed contrast enhancement (49.3%). Thus, among 152 enhanced lesions, 102 (65.4%) were mass, 4 (2.7%) were non-mass enhancements, and 46 (29.5%) corresponded to another kind of lesions such as cyst, Intramammary ganglion, among others. The kinetic curve for mass and non-mass enhancements was persistent in 87 lesions (82%), plateau in 16 (15.1%) and wash-out in three of them (2.8%). Table 4 summarizes the findings reported at MRI examinations. Most of them were nodules (52.27%) or simple cysts (27.9%). Additionally, nine associated findings were identified, as reported in Table 5.

**Table 3:** Main characteristics of MRI findings

Parameter	Results
*Background parenchymal enhancement*	
Minimal	12 (11.4%)
Mild	38 (36.2%)
Moderate	36 (34.3%)
Marked	19 (18.1%)
*Breast density*	
ACR A	3 (2.8%)
ACR B	21 (20.0%)
ACR C	30 (28.6%)
ACR D	51 (48.6%)
*Enhancement*	
Mass	102 (33.1%)
Non-mass	4 (1.3%)
Other	46 (14.9%)
Non-enhancement	156 (50.6%)
*Kinetic post-contrast curve*	
Persistent	87 (82%)
Plateau	16 (15.1%)
Wash-out	3 (2.8%)

**Table 4:** Summary of findings reported at MRI examinations

Lesion type	Quantity
Nodule	163
Simple cyst	92
Intramammary ganglion	10
Cluster of microcysts	10
Ductal ectasia	7
Fat necrosis	2
Hemorrhagic cyst	5
Complex cyst	5
Epidermal cyst	2
Inflammatory mass	1
Hamartomas	3
Mucinous lesion	1
Enhanced foci	1
Papilloma	1
Non mass enhancements	4
*Incidental findings*	
Skin compromise	1
Nipple retraction	1
Adenopathies	3
Venous malformations	1
Liver cysts	1
Desmoid tumor	1
Multiple hamartomas (Von Meyenburg complex)	1

**Table 5:** Ultrasonography and Mammography findings Vs DCE characterization of BI-RADS 3 patients

Appearance of ultrasound findings	DCE characterization
Ultrasound nodules: 112	
Circumscribed nodules: 103	Non-enhanced nodules: 44
	Persistent-enhanced nodules: 27
	Plateau-enhanced nodules: 4
	Wash-out nodules: 2
	Non-Mass Enhancement: 1
	Fat necrosis: 1
	Inflammatory ganglion: 1
	Hamartoma: 1
	Simple cysts: 8
	Enhanced foci: 1
	Without representation: 13
New nodules: 6	Non-enhanced nodules: 3
	Persistent-enhanced nodules: 2
	Without representation: 1
Nodules with enlargement: 3	Persistent-enhanced nodules: 2
	Without representation: 1
Simple cysts: 29	Simple cysts: 24
	Complex cysts: 2
	Without representation: 3
Complex cysts: 14	Non-enhanced solid nodules: 1
	Simple cysts: 9
	Hemorrhagic cyst: 1
	Complex cysts: 1
	Mucinous lesions: 1
	Without representation: 1
Ductal ectasia: 2	Simple cysts: 2
Clusters of microcysts: 8	Clusters of microcysts: 5
	Simple cysts: 2
	Without representation: 1
Asymmetry: 1	Without representation: 1
Epidermal cyst: 1	Epidermal cyst: 1
Findings detected only at MRI: 33	Non-enhanced nodules: 9
	Persistent-enhanced nodules:9
	Plateau-enhanced nodules: 2
	Clusters of microcysts: 1
	Simple cysts: 8
	Hemorrhagic cyst: 1
	Intramammary ganglion: 3

### MRI outcome of previous mammographic and ultrasonographic findings

The possible correlation between the MRI and mammography/ultrasonography findings for BIRADS 3 and 4A admitted studies are presented in Tables 5, 6, 7 and 8, respectively. A total of 282 findings were described in previous mammography and ultrasonography examinations. From them, 46 were not visualized at the MRI examinations. The most frequent mammographic and ultrasonography findings reported in BIRADS 3 were circumscribed solid nodules, multiple nodules, complicated cysts, and asymmetries; they were correlated at MRI with solid non-enhanced nodules, solid nodules with persistent enhancement, and cysts (Tables 5 and 6). The most frequent findings from previous BIRADS 4A were circumscribed nodules and multiple nodules, correlated with solid nodules with persistent enhancement (Tables 7, 8) at MRI.

**Table 6:** Mammography findings Vs DCE characterization of BI-RADS 3 patients

Appearance of mammography findings	DCE characterization
Mammographic nodules: 2	Non-enhanced nodules: 1
	Persistent-enhanced nodules: 1
Asymmetries: 11	Clusters of microcysts: 1
	Non-mass enhancement: 2
	Non-mass enhancement: 1
	Intramammary ganglion: 1
	Without representation: 6
Microcalcifications: 1	Without representation: 1
Findings detected only at MRI: 6	Persistent-enhanced nodules: 1
	Clusters of microcysts: 1
	Simple cysts: 3
	Intramammary ganglion: 1

**Table 7:** Ultrasonography findings versus DCE characterization of BI-RADS 4A patients

Appearance of ultrasound findings	DCE characterization
Ultrasound nodules: 63	
Circumscribed nodules: 54	Non-enhanced nodules: 16
	Persistent-enhanced nodules: 17
	Plateau-enhanced nodules: 6
	Wash-out nodules: 1
	Simple cysts: 5
	Hemorrhagic cyst: 1
	Hamartoma: 2
	Nodular fat tissue without enhancement: 1
	Without representation: 5
Nodules with enlargement: 6	Persistent-enhanced nodules: 4
	Plateau-enhanced nodules: 1
	Without representation: 1
New nodules: 3	Non-enhanced nodules: 2
	Complex cyst: 1
Simple cysts: 20	Simple cysts: 15
	Without representation: 5
Ductal ectasia: 4	Ductal ectasia: 2
	Ductal ectasia with proteinaceous content: 2
Intraductal papilloma: 1	Intraductal papilloma: 1
Complex cyst: 1	Complex cyst with plateau enhancement: 1
Asymmetries: 2	Without representation: 2
Findings detected only at MRI: 32	Persistent-enhanced nodules: 3
	Plateau-enhanced nodules: 2
	Clusters of microcysts: 2
	Simple cysts: 16
	Hemorrhagic cyst: 1
	Intramammary ganglion: 4
	Ductal ectasia: 3
	Fat necrosis: 1

**Table 8:** Mammography findings versus DCE characterization of BI-RADS 4A patients

Appearance of mammography findings	DCE characterization
Mammographic nodule: 6	Non-enhanced nodules: 1
	Plateau-enhanced nodules: 2
	Hemorrhagic cyst: 1
	Epidermal cyst: 1
	Without representation: 1
Asymmetry: 3	Without representation: 3
Segmental calcifications: 1	Without representation: 1
Findings detected only at MRI: 1	Multiple hamartomas: 1

On the other hand, 72 incidental MRI findings were identified, 39 in patients previously categorized as BIRADS 3 and 33 as BIRADS 4A. These were: 26 nodules, 27 simple cysts, 4 clusters of microcysts, two hemorrhagic cysts, eight intramammary ganglions, three ductal ectasias, one fat necrosis, and one von Meyenburg complex (multiple biliary hamartomas). However, none of these findings was identified as malignant.

**Fig. 2:**
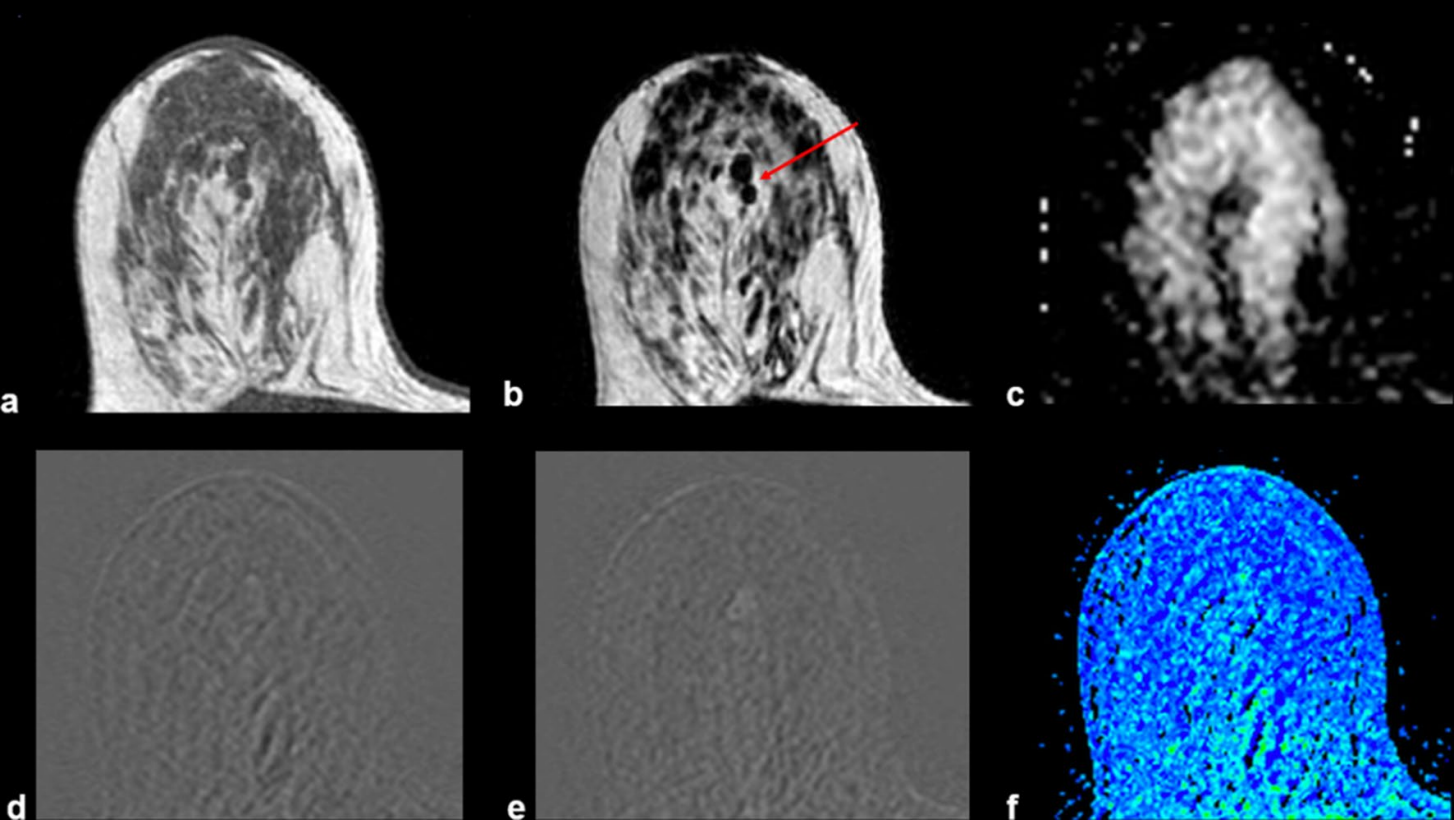
Example of MRI findings of a previous BIRADS 3 examination. A 63-year-old patient underwent follow-up of a periareolar solid nodule of 15 × 6 mm in the right breast classified as BIRADS 3 by previous examinations. Top: T1-W axial (**a**), T2 VISTA axial (**b**) and ADC map (**c**), which show a bilobed hypointense nodule in the union of the upper quadrants of the right breast with no restricted diffusivity. T1-W sequence shows a scattered glandular tissue classified as ACR D, occupying the four quadrants. Bottom: initial postcontrast subtraction (**d**) without enhancement and delayed subtraction (**e**) showing slight enhancement at the nodule location, which corresponds to the kinetic response described by the wash-in map (**f**). Thus, it was assigned BIRADS 2

**Fig. 3:**
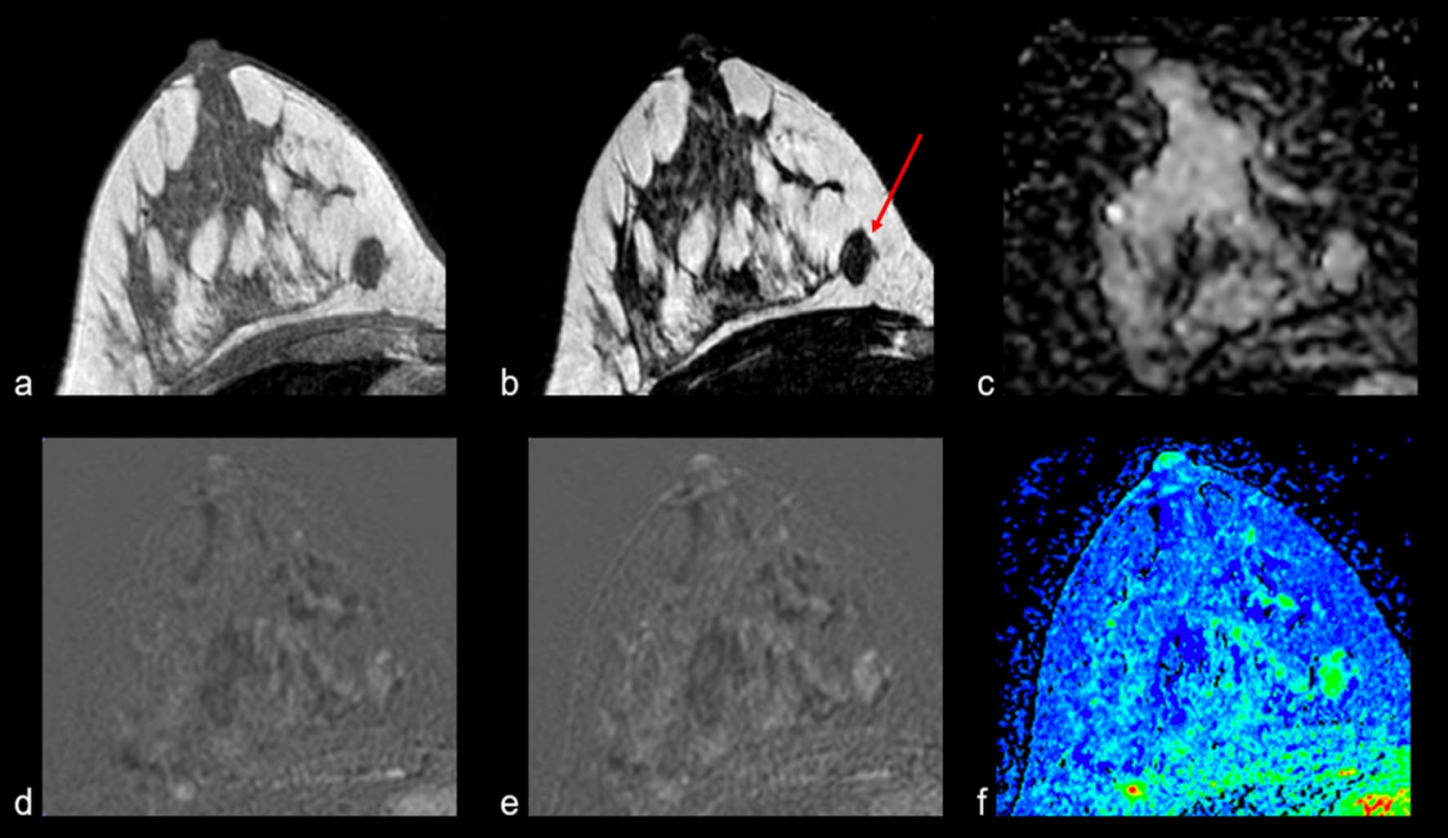
Example of MRI finding of a previous BIRADS 4A echography examination. A 28-year-old patient with recent-onset solid nodules in both breasts. Above: T1-W axial (**a**), T2 VISTA axial (**b**) and ADC map (**c**) show a hypointense mass with regular contours and an ADC value about 1.5 × 10^−3^mm^2^/s, indicative of a benign finding. Bottom: initial (**d**) and delayed (**e**) postcontrast subtractions demonstrated a persistent contrast enhancement corresponding to the kinetic response described by the wash-in map (**f**). Due to the characteristics of the lesion, it results in a BIRADS 2 lesion

**Fig. 4:**
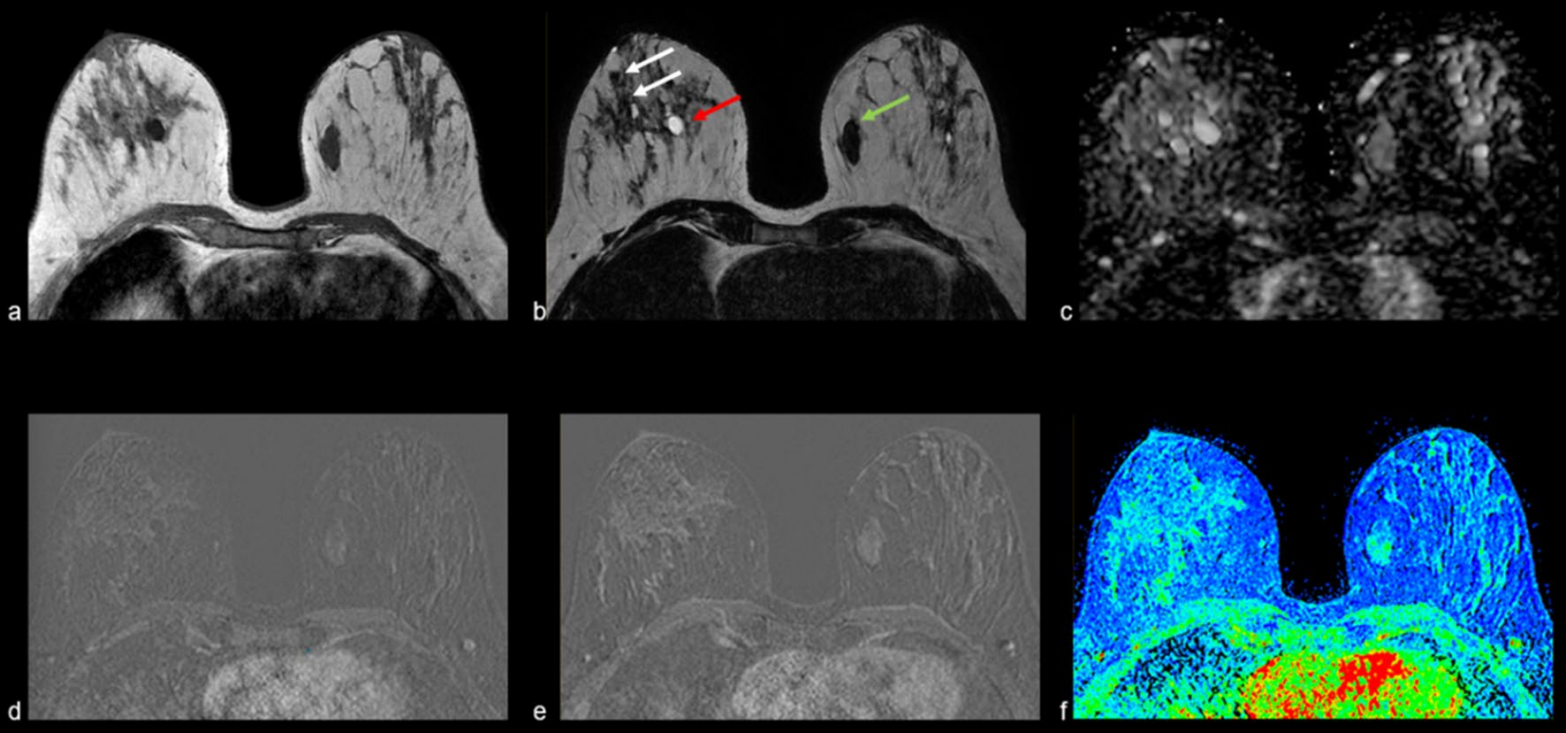
Example of MRI findings of breast lesions categorized as BIRADS 0 and BIRADS 3 by previous examinations. A 43-year-old patient with multiple bilateral findings, most of them simple cysts (white arrows). At the left breast (green arrow), a solid lobulated lesion, hypointense on T1W (**a**) and T2W (**b**) sequences, which shows an ADC (**c**) of 1.43 × 10^−3^mm^2^/s, with the contrast medium it shows an homogeneous wash-in pattern (**f**), with a slight progressive uptake from the initial (**d**) to delayed (**e**) postcontrast subtractions, corresponding to a benign lesion with characteristics of fibroadenoma. At the right breast (red arrow), a nodule, hypointense on T1W (**a**) and hyperintense on T2W (**b**), which shows no restricted diffusivity and no contrast enhancement. Both lesions were categorized as BIRADS 2

**Fig. 5:**
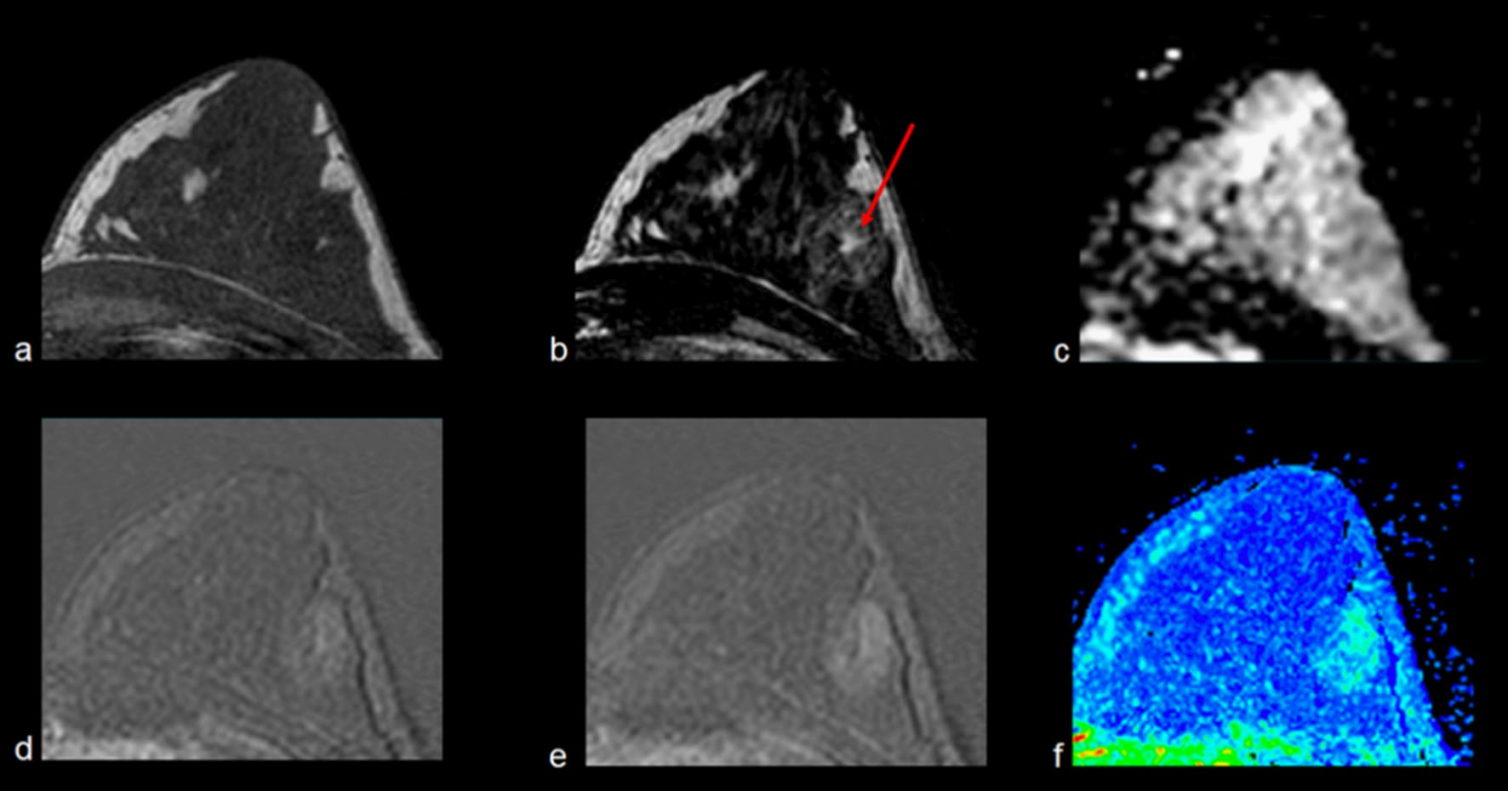
Example of MRI findings of breast lesions categorized as BIRADS 4A by previous echography. A 27-year-old patient with a family history of breast cancer in mother and grandmother. Above: T1-W axial (**a**), T2 VISTA axial (**b**) and ADC map (**c**) show a heterogeneous mass with a solid component in the periphery and a hyperintense central area due to a fatty component, no restricted diffusivity with an ADC of 1.9 × 10^−3^mm^2^/s. Bottom: initial (**d**) and delayed (**e**) postcontrast subtractions, and the wash-in map (**f**) show a progressive uptake of contrast. Morphological and kinetic features are compatible with hamartoma reported in previous echography. It was assigned as the BIRADS-2 category

**Fig. 6:**
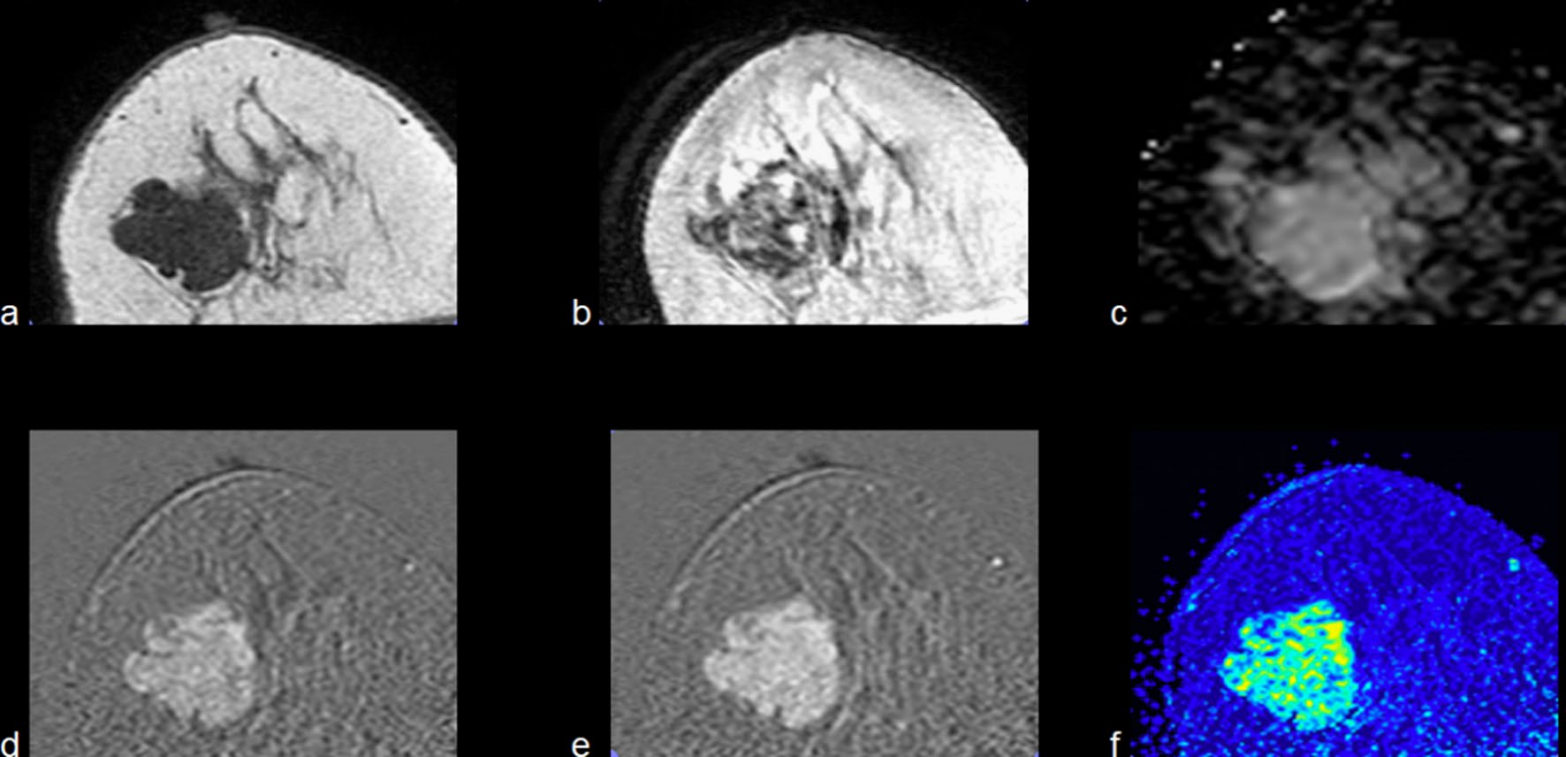
Example of MRI finding of breast lesion classified as BIRADS 4A by echography. A 63-years-old patient presents a solid lobulated mass, hypointense on T1W (**a**) with no diffusivity restriction (**c**), which shows a periphery with cystic content on T2W (**b**). The contrast uptake shows an ascending and plateau curve from the initial (**d**) to delayed (**e**) subtractions, with a homogeneous gradual wash-in (**f**). Those features indicate benignity (Kaiser score 2); however, it was classified as BIRADS 4 at MRI due to mass size increase. The pathological analysis confirmed it as a fibroadenoma

**Fig. 7:**
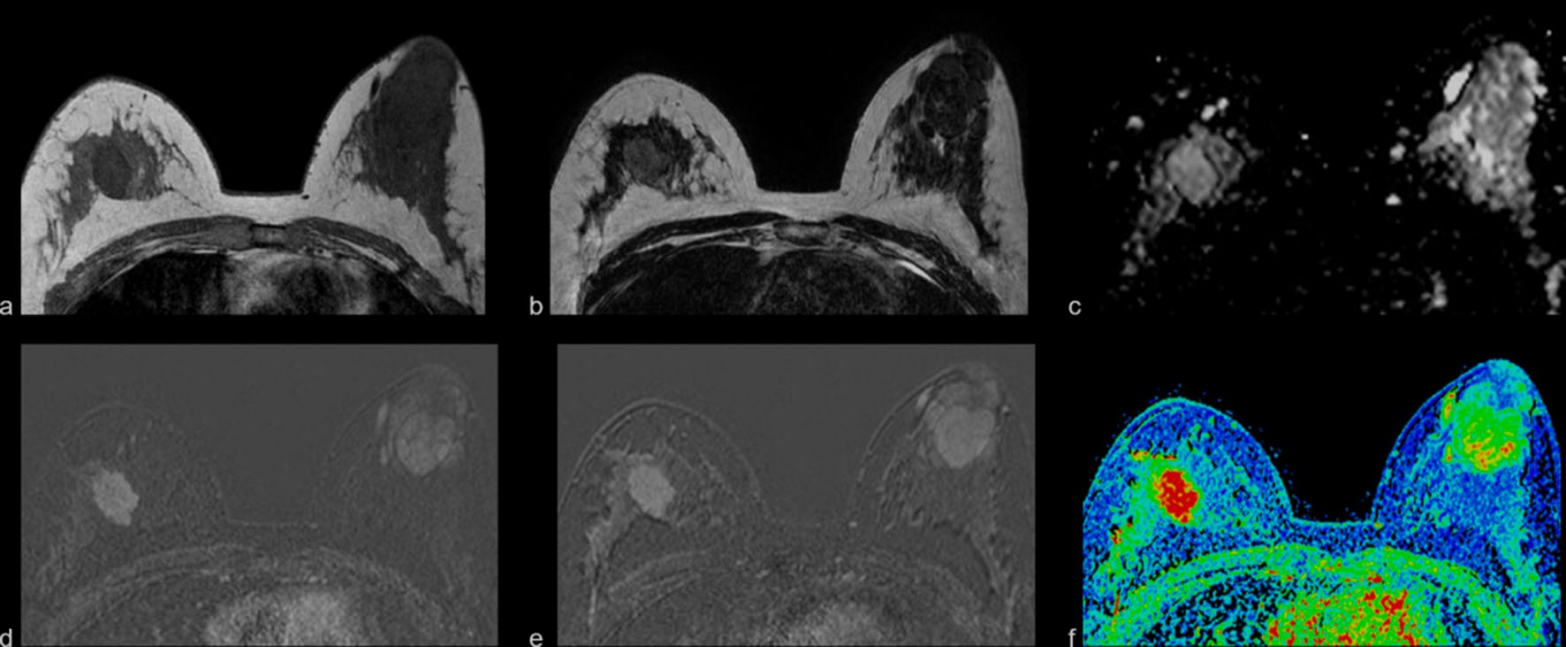
Example of MRI finding of breast lesion classified as BIRADS 4A by echography. A 26-years-old patient with a giant fibroadenoma at the left breast without suspicious contrast enhancement and size stability compared to previous ultrasound studies. At the right breast, a mass with lobulated contours, hypointense on the T1W sequence (**a**), which shows hyperintensity to the glandular parenchyma on the T2 sequence (**b**), and an ADC (**c**) of 1.8 × 10^−3^mm^2^/s. Postcontrast subtraction images show a contrast enhancement that describes an ascending and plateau uptake from initial (**d**) to delayed (**e**) subtractions, with rapid enhancement (**f**). It presented characteristics of fibroadenoma; however, as it has shown a size increase compared to previous ultrasonography studies, histological analysis was required, resulting in a pericanalicular fibroadenoma

**Fig. 8:**
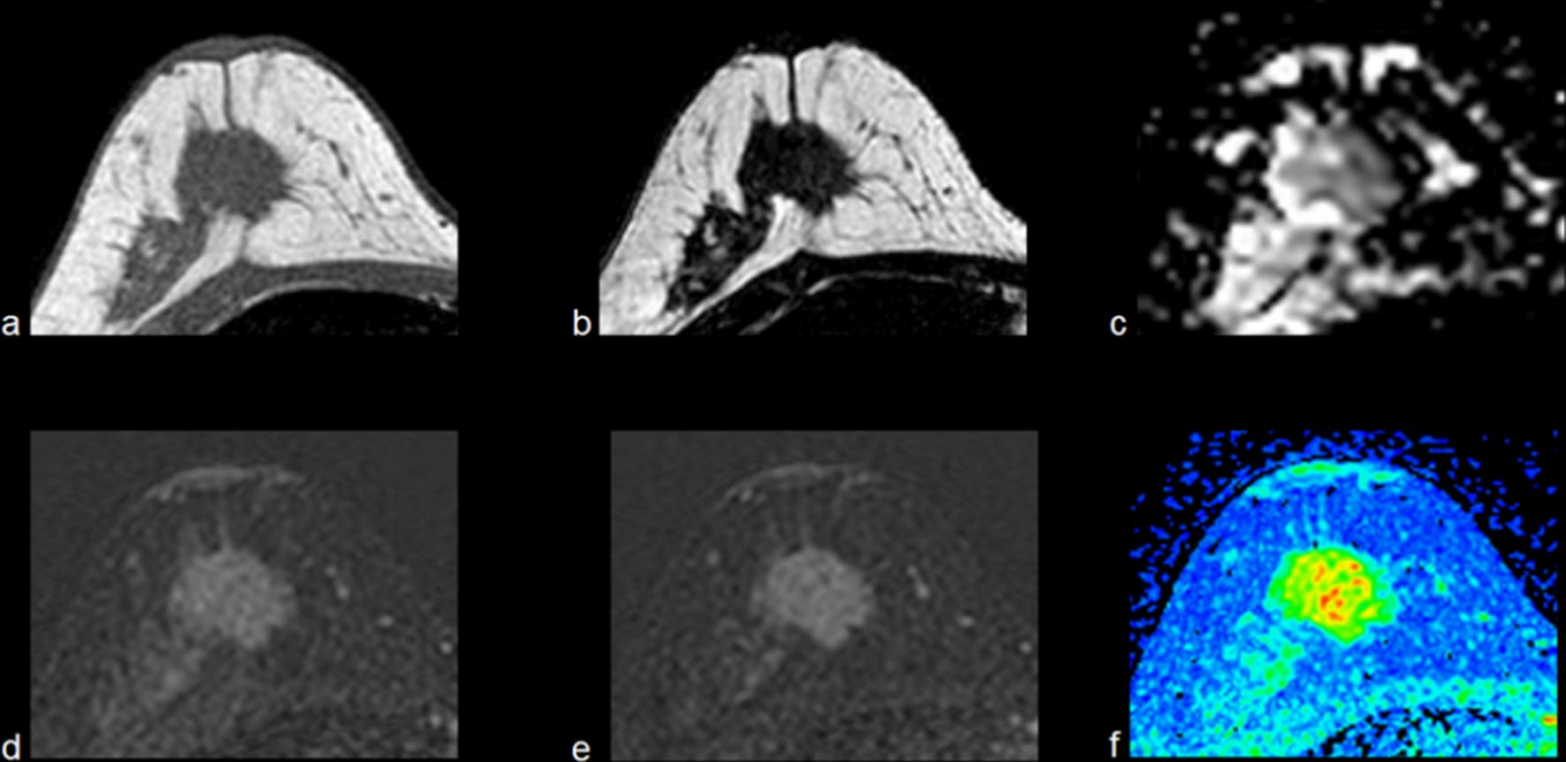
Example of MRI finding of breast lesion classified as BIRADS 4A by echography. A 44-years-old patient with a solid mass with irregular contours at the right breast presents superior external spiculations. It appears hypointense on T1W (**a**) and T2W (**b**), and ADC (**c**) value of 0.8 × 10^−3^mm^2^/s. At initial (**d**) and delayed (**e**) postcontrast subtraction images, it shows a homogeneous gradual enhancement (**f**) with a wash-out pattern. Thus, it was classified as BIRADS 5. The pathological analysis confirmed it as Invasive Ductal Carcinoma

Figures 2, 3, 4, 5, 6, 7 and 8 present MRI images examples of representative findings categorized as BIRADS 3 or 4A by previous mammography or ultrasonography examinations. Figure 2 presents the most typical and easy-decision case; it is a non-enhancing lesion (Kaiser Score 0 / BIRADS 2). Among 184 nodules reported in the previous examinations of recruited patients, 67 (35%) did not show contrast enhancement; thus, these lesions were downgraded to BIRADS 2 i.e., benign lesions. Figures 3 and 4 show examples of some contrast-enhanced lesions with circumscribed margins, persistent signal enhancement time curves, and no restricted diffusivity, features compatible with benign findings (BIRADS-2). Figure 4. Illustrates the advantage of MRI scans in presence of multiple findings; 3D analysis and contrast enhancement are useful in those cases. Figure 5 shows an example of hamartoma, a benign lesion that presents varying amounts of benign epithelial components, fibrous tissue, and fat tissue. Figures 6 and 7 show examples of false positives cases, i.e., benign lesions categorized as BIRADS 4. Two cases describing imaging features of low risk of malignancy (Kaiser score 2) but were biopsy recommended due to size increasing of the lesions. Finally, Fig. 8 shows an example of a malignant, categorized as BIRADS 5, which presents irregular contours with superior external spiculations and a homogeneous enhancement, with a wash-out pattern and ADC value of 0.8 × 10^−3^mm^2^/s.

### BIRADS re-categorization

Among 105 patients, 71 (67.62%) were admitted for having a previous BIRADS 3 imaging examination, and 34 (32.38%) for having BIRADS 4A. Table 9 presents the mammographic and ultrasonographic findings that determined the BIRADS examination category and their respective classification on the MRI examination. Although 282 findings were described in the previous examinations, 131 were decisive for establishing the BIRADS category: 88 from BIRADS 3 and 43 from BIRADS 4A examinations. The mammographic findings that led to a BIRADS 3 categorization were asymmetries (*n* = 9), single nodules (*n* = 4), multiple nodules (*n* = 1), and microcalcifications (*n* = 1), while the ultrasonography findings were: solid nodule (*n* = 36), multiple nodules (*n* = 17), complicated cyst (*n* = 13), accumulation of microcysts (*n* = 5), ductal ectasia (*n* = 1) and asymmetry (*n* = 1). On the other hand, for BIRADS 4A patients, the mammographic findings were asymmetry (*n* = 2), nodule (*n* = 3), multiple nodules (*n* = 2), and segmental calcifications (*n* = 1), and the ultrasonography findings were solid nodules (*n* = 27), multiple nodules (*n* = 1), ductal ectasia (*n* = 3), asymmetry (*n* = 2), complex cyst (*n* = 1) and intraductal papilloma (*n* = 1).

**Table 9:** BI-RADS categorization at MRI of previous mammographic and ultrasound findings

	Findings at previous examinations	BI-RADS at MRI				
		1	2	3	4	5
BI-RADS* 3						
*Mammographic findings*						
Asymmetry	9	2	6	1		
Nodule	4	1	3			
Multiple nodules	1		1			
Microcalcifications	1				1	
*Ultrasound findings*						
Solid nodule	36	2	30	1	3	
Multiple nodules	17		15	1	1	
Complicated cyst	13		13			
Cluster of microcysts	5		5			
Ductal ectasia	1		1			
Asymmetry	1		1			
*BI-RADS 4A*						
*Mammographic findings*						
Asymmetry	2	1	1			
Nodule	3		2		1	
Multiple nodules	2	1	1			
Segmental calcifications	1	1				
*Ultrasound findings*						
Solid Nodule	27	1	15	3	5	3
Multiple nodules	1		1			
Ductal ectasia	3		1	1	1	
Complicated cyst	1	1				
Intraductal papilloma	1		1			
Asymmetry	2		2			

*Breast imaging-reporting and database system

Among 71 patients included as category BIRADS 3, 64 (90.14%) were re-classified on category BIRADS 1 or 2, which means that they can continue the conventional biannual screening; 3 continued in category BIRADS 3, but they have remained stable in the follow-ups, and four were upgrades to BIRADS 4 and underwent percutaneous biopsy, all of them resulting in fibroadenoma. Additionally, among 34 patients previously categorized as BIRADS 4A, 22 (64.7%) were recategorized as BIRADS 1 or 2; four (11.76%) as BIRADS 3, who have remained with stable lesions in follow-up; five (14.7%) remained as BIRADS 4, with benign histological analyzes (two fat necrosis, two fibroadenomas, and one canalicular fibroadenoma); and three (8.8%) patients were recategorized as BIRADS 5, these with malignant histology.

Finally, 86 (81.9%) subjects were downgraded to BIRADS 1 or 2, which means that 64.7% (22) biopsies and 90.1% (64) short-term follow-ups were avoided. Eight patients (7.61%) were categorized as BIRADS 3 and considered negative cases due to no changes after short-term follow-up; 9 (8.41%) subjects were categorized as BIRADS 4 (three of them upgraded from BIRADS-3 lesions), and 3 were upgraded to BIRADS 5 (2.8%). However, only the three BIRADS 5 lesions were breast cancer diagnosed (Invasive Ductal Carcinoma), and the nine BIRADS 4 as benign lesions.

### Diagnostic performance

**Fig. 9:**
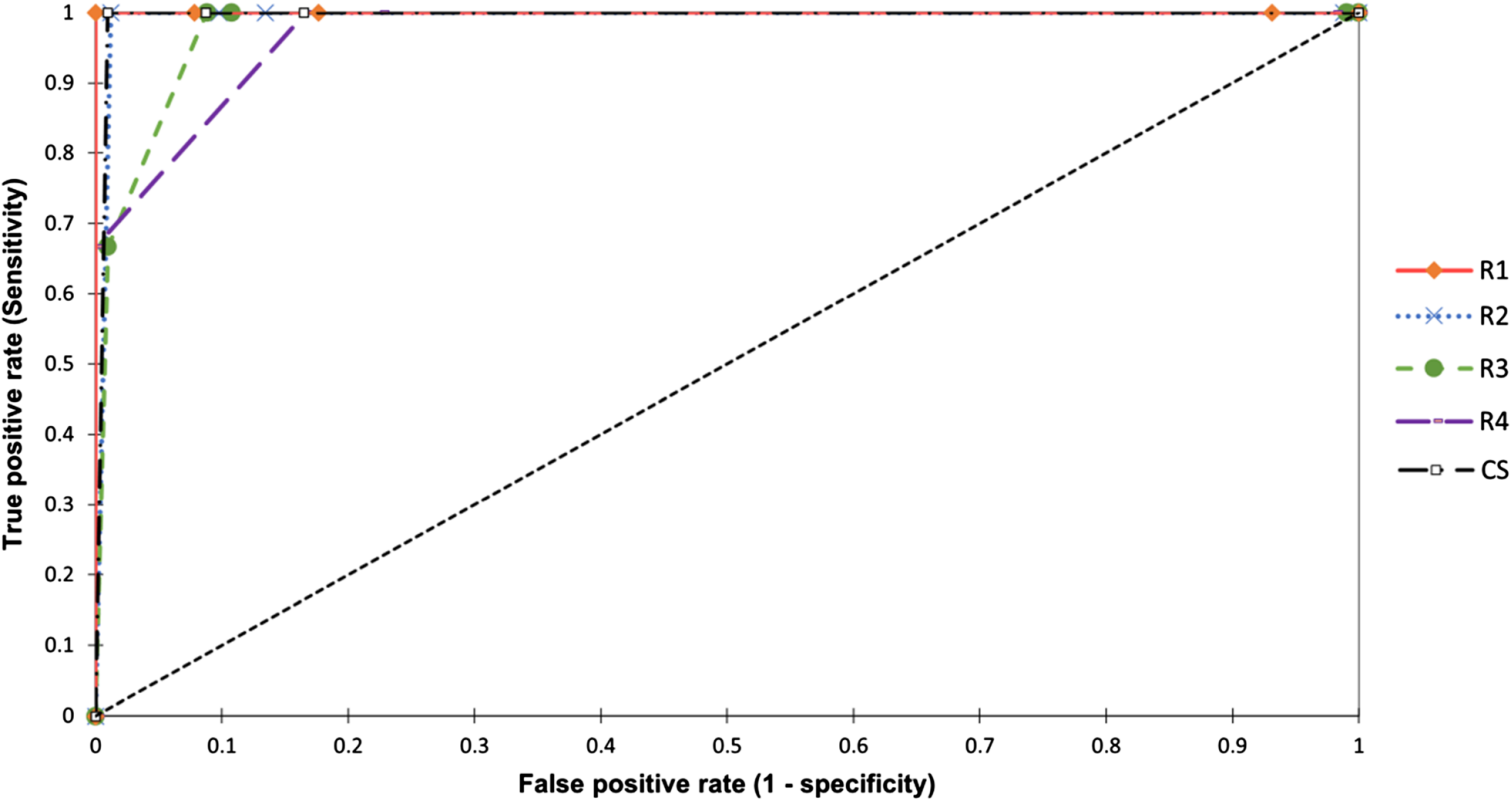
Receiver operating characteristic curves (ROC) for each radiologist and the consensual decision (CS)

Considering BI-RADS 3 to 5 as positive findings and 1 and 2 as negative, Fig. 9 presents the receiver operating characteristic curves (ROC) for each radiologist and the consensual decision (CS). Likewise, Table 10 presents the area under the roc curve (AUC), sensitivity, specificity, and positive and negative predictive values. For the consensual decision, ROC analysis revealed an area under the ROC curve of 0.995 (95% CI: 0.986–1.00), three true-positive, 94 true negatives, 9 false positives, and no false-negative cases. Eight false positives were categorized as BI-RADS 4, and the other eight as BI-RADS 3. The sensitivity, specificity, positive and negative predictive values were calculated as 100%, 83.5%, 10.5%, and 100%, respectively. Note that the small number of positive cases limits the statistical analysis of sensitivity and PPV results.

**Table 10:** MRI diagnostic performance for the four radiologists and the consensual decision (CS)

	AUC*	CI** 95%	Sensitivity	CI 95%	Specificity	CI 95%	PPV***	NPV***
R1	1000	(1.00–1.00)	1.00	(0.380–1.00)	0.824	(0.737–0.886)	0.143	1.000
R2	0.994	(0.982–1.00)	1.00	(0.380–1.00)	0.866	(0.773–0.925)	0.214	1.000
R3	0.980	(0.949–1.00)	1.00	(0.380–1.00)	0.892	(0.815–0.940)	0.214	1.000
R4	0.972	(0.918–1.00)	1.00	(0.380–1.00)	0.775	(0.638–0.845)	0.115	1.000
CS	0.995	(0.986–1.00)	1.00	(0.289–1.00)	0.835	(0.750–0.895)	0.105	1.000

*Area under ROC curve

**Confidence interval

***Positive predictive values

****Negative predictive values

Regarding radiologist experience, AUC increase from less (R4) to most (R1) experienced radiologist. Four radiologists identify the three malignant lesions, although R3 and R4 assigned a category BIRADS 4 to two of them. Specificity was significantly lower for the less experienced radiologist (R4); however, the most experienced was not the best performer. A detailed review of the results shows that she assigned BIRADS 3 category to some studies that presented image characteristics compatible with benignity but presented some differences with the findings reported in previous studies.

### Interobserver agreement

Table 11 presents the generalized kappa and the AC1 between radiologists. As was expected, the AC1 statistics always lead to higher values than kappa statistics. According to Landis and Koch’s benchmark scale [29], the overall interobserver agreement given by the kappa coefficient of 0.207 indicated a fair agreement; however, AC1 showed a substantial agreement (0.65). Best concordance was observed between the two radiologists with intermediate experience (R2, R3: 0.80), while the greatest differences were reported between the two radiologists with the major experience and the one with the least (R1, R4: 0.56 and R2, R4: 0.57); however, moderate, and substantial agreements were obtained for all cases.

**Table 11:** Inter-observer agreement in the assignation of BI-RADS category to MRI studies

Radiologist	Fleiss Kappa			Gwet AC1**		
	Kappa	*P* value	CI* 95%	AC1	*P* value	CI 95%
R1,R2,R3,R4	0.20789	7.20E−03	(0.058,0.358)	0.65246	0	(0.603,0.784)
R1,R2,R3	0.30007	1.03E−03	(0.125,0.475)	0.69343	0	(0.566,0.754)
R2,R3,R4	0.20888	0.01814	(0.037,0.381)	0.66	0	(0.566,0.754)
R1,R2	0.30882	6.02E−03	(0.091,0.526)	0.66017	0	(0.536,0.784)
R1,R3	0.23186	0.0209	(0.036,0.428)	0.68642	0	(0.584,0.789)
R1,R4	0.07758	0.3919	(− 0.102,0.257)	0.56142	2.22E−16	(0.448,0.675)
R2,R3	0.36596	0.0034	(0.124,0.608)	0.80082	0	(0.701,0.901)
R2,R4	0.10859	0.26947	(− 0.086,0.303)	0.57269	9.88E−13	(0.44,0.706)
R3,R4	0.18008	0.07591	(− 0.019,0.379)	0.70786	0	(0.609,0.807)

*Confidence Interval

**First-order agreement coefficient

## Discussion

BIRADS 3 and 4A lesions are known to have a small probability of being malignant (less than 2% and 10%, respectively). In order to exclude malignancy, BIRADS 3 lesions are short followed, while BIRADS 4A undergo percutaneous biopsy. It can result in many unnecessary exams, but it could also delay the detection of malignant lesions.

Hence, in recent years, diagnostic methods such as tomosynthesis, elastography, contrast mammography, and magnetic resonance imaging have been proposed, combined with mammography and ultrasonography, to reduce diagnostic uncertainty, save follow-ups and avoid morbid and expensive invasive procedures [30, 31]. Breast MRI examinations that include dynamic contrast-enhanced sequences allow the evaluation of the kinetic and morphological characteristics of the lesions, which cannot be adequately characterized by ultrasonography or mammography, proving to be useful for differentiating benign from probably malignant lesions.

In this study, breast MRI findings of 105 patients, with previous mammography or ultrasonography examinations classified as BIRADS 3 or 4A were compared. Histopathology revealed three invasive ductal carcinoma lesions, resulting in a prevalence of malignancy of 2.8%. The sensitivity of breast-MRI was 100%, the specificity was 83.5% (75.0–89.5%), PPV was 10.5%, and NPV was 100%. The small number of positive cases limits the statistical analysis and the power of sensitivity results. However, as this study focused on determining whether the breast MRI examination allows excluding malignancy for reducing unnecessary biopsies or short follow-ups, we compute the power sample size for specificity. It was 80%, with a significance level of 0.05, a core needle biopsy specificity of 0.98 [32] and a breast MRI specificity of 0.85, according to previous studies for inconclusive mammography or ultrasonography cases (62.4–97%) [10, 18, 33–37]. In this study, BIRADS category 3 was defined as a positive finding due to that it remains a small risk of malignancy (less than 2%), which generates lower specificity concerning the obtained if these cases were considered negative or benign results, as in other studies [33, 35, 37].

Regarding detected lesions, among 282 findings reported at previous imaging examinations, 120 (41.6%) were identified as benign by their kinetic characteristics; 67 of them (55.8%) due that they do not enhance with the contrast medium; and present morphological characteristics that allow characterizing it as benign findings such as asymmetries, cysts, and ductal ectasias. On the other hand, breast MRI was useful in diagnosing three invasive ductal carcinomas, categorized as BIRADS 5, which would prioritize the percutaneous biopsy performing, enhancing the diagnosis and treatment opportunity for these patients. Additionally, MRI helped identify other breast and associated lesions not previously visualized in the other imaging modalities. Although none of them turned out to be a malign finding, this shows the ability of MRI to identify lesions hidden for other modalities. Nevertheless, MRI fails to represent several findings described from other imaging examinations, including microcalcifications observed in mammography. It seems to indicate that MRI may help resolve indeterminate cases in screening with other imaging techniques; however, its use as an individual screening method was outside the scope of this study.

Altogether, malignancy was excluded in 86 patients (81.9%), avoiding 22 (64.7%) of unnecessary biopsies and 64(90.1%) of short-term follow-ups. Three BIRADS 3 lesions (4.2%) were upgraded to BIRADS 4, causing unnecessary immediate biopsies; four BIRADS 4 (11.7) were downgraded to BIRADS 3, which implies a reduction in the number of unnecessary biopsies, but a delay in the diagnostic conclusion. Three BIRADS 4 patients (8.82%) were upgraded correctly to BIRADS 5. Therefore, as was previously reported [36–38], in this study, MRI showed to be very useful for downgrading the BIRADS category from indeterminate (BIRADS 3 or 4A) to normal or benign findings (BIRADS 1 or 2). It entails physical and psychological benefits to patients and savings for health systems by increasing the time for breast imaging follow-up and preventing unnecessary percutaneous biopsies.

On the other hand, although differences between radiologists were not statistically significant, this study showed a greater concordance between the findings reported by radiologists who are experts in the interpretation of breast MRI examinations than those with less experience. Specifically, the radiologist with less experience has greater difficulties in distinguishing benign findings, even when the Kaiser score was used, as was reported in previous studies [24].

This study has some limitations. First, only those examinations categorized as BIRADS 4 or 5 at MRI have undergone a biopsy because it was considered unnecessary to biopsy on lesions that showed characteristics of benignity at MRI, i.e., BIRADS 1 to 3. In those cases, the reference standard was the short follow-up result, which is considered an imperfect standard that could bias the diagnostic performance. Second, the number of included patients is not extensive; it does not include enough cases of interest, such as patients with microcalcifications or architectural distortion. One study with those specific characteristics could establish the effect of using MRI to solve indeterminate results in those cases. Third, the Kaiser score flowchart was not systematically carried out, and either was it recorded. It avoided to evaluating their effect on the reported results. It has been shown to be relevant in recent studies [39–41], which should be considered in future works. And fourth, the cost-effectiveness of the MRI relative to continue with the management defined by previous examinations was not calculated, due precisely to the first limitation described above. It is important because one of the main barriers to implementing MRI in diagnosing breast cancer is its high cost. However, our results allow us to suppose that the abbreviated protocols, recently proposed in the literature [42, 43], could be equally useful in these cases. Because the BIRADS category of most of the findings was resolved by morphological analysis or by the absence of contrast enhancement, which can be observed in early sequences of DCE, the use of abbreviated protocols could improve the cost-effectiveness of using MRI as a problem-solving tool in breast cancer screening.

In conclusion, this study shows the feasibility of using Breast MRI to clarify the interpretation of lesions classified as BIRADS3 and 4A on conventional medical imaging, i.e., mammography and ultrasonography. It can downgrade up to 86% of lesions for excluding malignancy, which could avoid unnecessary biopsies and short-term follow-ups in a substantial number of cases.

## Data Availability

The datasets used and/or analyzed during the current study are available from the corresponding author on reasonable request.

## Abbreviations

3D: Three-dimensional
AC1: First-order agreement coefficient
ACR: American College of Radiology
ADC: Apparent diffusion coefficient
AUC: Area under ROC curve
BIRADS: Breast Imaging-Reporting and Database System
CAD: Computer-aided diagnosis
CI: Confidence Interval
DWI: Diffusion-weighted images
FOV: Field-of-view
IATM: Instituto de Alta Tecnología Médica
MRI: Magnetic resonance imaging
NPV: Negative predictive values
PPV: Positive predictive values
ROC: Receiver operating characteristic
SPIR: Spectral presaturation with inversion recovery
STIR: Short tau inversion recovery
THRIVE: T1W high-resolution isotropic volume examination
VISTA: 3D volume isotropic turbo spin-echo acquisition (Philips)
